# GRP78 Promotes Neural Stem Cell Antiapoptosis and Survival in Response to Oxygen-Glucose Deprivation (OGD)/Reoxygenation through PI3K/Akt, ERK1/2, and NF-*κ*B/p65 Pathways

**DOI:** 10.1155/2018/3541807

**Published:** 2018-04-10

**Authors:** Qian Liu, Yun Li, Lin Zhou, Yunzi Li, Pengfei Xu, Xiaoyun Liu, Qiushi Lv, Juanji Li, Hongquan Guo, Haodi Cai, Rui Sun, Xinfeng Liu

**Affiliations:** ^1^Department of Neurology, Jinling Hospital, Medical School of Nanjing University, 305 East Zhongshan Road, Nanjing, Jiangsu 210002, China; ^2^College of Chemistry and Materials Science, Jiangsu Key Laboratory of Biofunctional Materials, Nanjing Normal University, Nanjing, Jiangsu 210023, China; ^3^Department of Neurology, Jinling Hospital, Southern Medical University, Nanjing, Jiangsu 210002, China; ^4^Department of Neurology, Jinling Hospital, Southeast University, Nanjing, Jiangsu 210002, China

## Abstract

When brain injury happens, endogenous neural stem cells (NSCs) located in the adult subventricular zone (SVZ) and subgranular zone (SGZ) are attacked by ischemia/reperfusion to undergo cellular apoptosis and death before being induced to migrate to the lesion point and differentiate into mature neural cells for damaged cell replacement. Although promoting antiapoptosis and NSC survival are critical to neuroregeneration, the mechanism has yet been elucidated clearly. Here in this study, we established an *in vitro* oxygen-glucose deprivation (OGD)/reoxygenation model on NSCs and detected glucose-regulated protein 78 (GRP78) involved in apoptosis, while in the absence of GRP78 by siRNA transfection, OGD/reoxygenation triggered PI3K/Akt, ERK1/2, and NF-*κ*B/p65 activation, and induced NSC apoptosis was attenuated. Further investigation, respectively, with the inhibitor of PI3K/Akt or ERK1/2 demonstrated a blockage on GRP78 upregulation, while the inhibition of NF-*κ*B rarely affected GRP78 induction by OGD/reoxygenation. The results indicated the bidirectional regulations of GRP78-PI3K/Akt and GRP78-ERK1/2 and the one-way signalling transduction through GRP78 to NF-*κ*B/p65 on NSC survival from OGD/reoxygenation. In conclusion, we found that GRP78 mediated the signalling cross talk through PI3K/Akt, ERK1/2, and NF-*κ*B/p65, which leads to antiapoptosis and NSC survival from ischemic stroke. Our finding gives a new evidence of GRP78 in NSCs as well as a new piece of signalling mechanism elucidation to NSC survival from ischemic stroke.

## 1. Introduction

Neural stem cells (NSCs), located in the embryonic brain, adult subventricular zone (SVZ), and subgranular zone (SGZ), are a type of self-renewing and multipotent cells that potentially produce diversity of mature neurons, astrocytes, and oligodendrocytes in response to either physiological development or pathological stimulation [1]. In adult brain pathologies, the induced NSCs in SVZ or SGZ are expected to migrate to the lesion point and differentiate into mature neurons and glial cells for damaged cell replacement [2, 3]. However, before inducing differentiation, a majority of these NSCs are directed to apoptosis and cell death by cellular hypoxia, energy exhaustion, oxidative stress, inflammation, and immune reaction derived from tissue ischemia and reperfusion [2, 4]. Since the involvement of massive signalling pathways in NSC apoptosis, exploring the undergoing mechanisms would provide strategies for NSC survival and neuroregeneration post ischemic stroke.

Accumulating researches have categorized endoplasmic reticulum (ER) stress response as one of the underlying mechanisms of cell apoptosis [5]. ER is a type of organelle existing in most eukaryotic cells, structured as a sac or tube-like cisterna [6]. The primary function of ER is to properly fold and facilitate the newly synthesized proteins and transport them to the Golgi apparatus through vesicles. In some pathologies, the folding load enormously increases and the unfolded and misfolded proteins accumulate in ER, leading to an unfolded protein response (UPR), which is documented as ER stress. ER stress emerges when disturbance occurs such as redox regulation, and the unfolded protein accumulation eventually leads to cell dysfunction and apoptosis. Therefore, ER stress response is a potential cause of cell death in ischemia, oxidative stress, and other damage [6–10].

There are a series of chaperone proteins located in ER which help to facilitate protein folding and transportation, among which glucose-regulated protein 78 (GRP78) plays a critical role in ER function and stress [11]. GRP78 is also known as heat shock 70 protein 5 (HSPA5) or binding immunoglobulin protein (BiP), which is coded by the HSPA5 gene [12]. As a molecular chaperone of the HSP70 family, GRP78 has been documented to react with a series of signalling pathways to release ER stress and rescue cells from apoptosis [13, 14]. However, whether ER stress induced apoptosis in NSCs or the GRP78-involved signalling mechanism by ischemia/reperfusion in neural cells is yet to be clearly elucidated.

In this study, to address the essential role of GRP78 in NSC apoptosis against ischemic stroke, we established the *in vitro* oxygen-glucose deprivation (OGD)/reoxygenation model on NSCs, investigated the induced NSC apoptosis, and discussed the signalling cross talk between GRP78 and the apoptosis-related signalling pathways. The results indicated the bidirectional regulations of GRP78-PI3K/Akt and GRP78-ERK1/2, as well as one-way signalling through GRP78 to NF-*κ*B/p65. The signalling cross talk leads to antiapoptosis and NSC survival from OGD/reoxygenation. Our finding has given a new evidence of ER stress on NSC apoptosis and a new piece of signalling mechanism elucidation to NSC survival from ischemic stroke.

## 2. Material and Methods

### 2.1. Animal and Reagents

The embryonic C57BL/6 mice used in this study were provided by the Model Animal Research Centre of Jingling Hospital (Nanjing, Jiangsu, P.R. China). This study has been approved by the Jinling Hospital Research Ethics Committee. The pregnant C57BL/6 mice were housed in a temperature-controlled environment (22 ± 0.5°C) with a 12 h light-dark cycle and allowed free access to food and water. All efforts were made to minimize animal suffering and reduce the number of animals used.

DMEM/F12 medium, B27 supplement, EGF, bFGF, penicillin/streptomycin, Accutase, poly-D-lysine (PDL), Lipofectamine™ 2000, and other reagents used in this study for NSC culture were purchased from Thermo Fisher Scientific (San Jose, CA, USA). Paraformaldehyde (PFA), Triton X-100, DMSO, LY294002, SCH77298, JSH23, and MTT for NSC treatments were purchased from Sigma-Aldrich (St. Louis, MO, USA). GRP78 siRNA (m2) sc-44303 for gene transfection was purchased from Santa Cruz Biotechnology Inc. (Dallas, TX, USA). All primary antibodies and HRP conjugate secondary antibodies used in this study were purchased from Cell Signalling Technology (Danvers, MA, USA). The PVDF membrane was purchased from Millipore (OH, USA). Goat anti-Rabbit/Mouse IgG (H+L) Alexa Fluor® 488/594 antibodies were purchased from Thermo Fisher Scientific (San Jose, CA, USA). Hoechst 33342/PI and DCFH-DA probe kits were purchased from Beyotime Biotech (Haimen, Zhejiang, China).

### 2.2. NSC Culture and Treatments

The NSCs were harvested from embryonic C57BL/6 mouse brain (E14), briefly as we described in our previous reports [15]. The neurosphere culture was kept in DMEM/F12 medium containing B27, EGF, and bFGF, at 37°C, 5% CO_2_. The medium was changed every three days. The passage was performed when the neurospheres grew to 50–100 *μ*m in diameter. The neurospheres were digested to a single-cell suspension and plated on a PDL-precoated surface depending on further tests. As long as NSCs are attached, the culturing medium was changed with DMEM/F12 in the absence of B27, EGF, and bFGF for cell starvation.

For signalling inhibitor pretreatment, after the cell starvation for 2 hours, 20 *μ*M LY294002, 10 nM SCH77298, and 8 *μ*M JSH-23 (final concentration) dissolved in DMSO-DMEM/F12 medium were administrated, respectively, to the monolayer NSC culture for 1 h, before OGD/reoxygenation induction. DMSO was used as the cosolvent, with the final concentration of 0.1%. The DMEM/F12 medium containing 0.1% DMSO was used as the vehicle control.

### 2.3. NSC OGD/Reoxygenation Induction

OGD/reoxygenation administration was applied on NSCs as we reported previously [16], with slight modification. In brief, the monolayer NSCs following starvation and signalling inhibitor treatments were transferred into a deoxygenated, glucose-free HBSS buffer. The culture was then incubated in an oxygen-deprived box aerated with 5% CO_2_ and 95% N_2_, at 37°C for 2 hours to model the OGD-injured NSCs. Ischemic NSCs were then transferred back into the DMEM/F12 medium and incubated at 37°C with 5% CO_2_ for 2, 4, 8, 12, and 24 hours, for further reoxygenation induction. The starved NSCs were incubated within HBSS with glucose at 37°C, 5% CO_2_, for 2 hours, followed by oxygen and glucose re-treatment, as a negative control.

### 2.4. siRNA Transfection

siRNA transfection was applied to knock down the protein expression of GRP78, following the manufacturer's instructions of GRP78 siRNA. Briefly, the monolayer culture of NSCs was cultured in the medium containing the Lipofectamine 2000-siRNA complex for 5 h and subjected to the inhibitor and OGD/reoxygenation treatments. The protein expression of GRP78 in the NSCs treated with siRNA transfection and OGD/reoxygenation treatment was detected to confirm the transfecting efficiency.

### 2.5. Cellular Detection

Cell viability was detected with MTT assay and Hoechst 33342/PI double staining as previously described [15, 17]. Cellular ROS production was detected with the DCF-DA probe kit, according to the manufacturer's instruction.

### 2.6. Western Blotting

The whole cell or nuclear lysate of the treated NSCs was collected for Western blotting. The samples were subjected onto 4–12% SDS-PAGE gel for electrophoresis as previously described [15]. The protein bands were transblotted onto the PVDF membrane, followed by blocking in 5% BSA-TBST buffer and incubation with the primary and HRP-conjugated secondary antibodies in sequence. The antigen-antibody complexes were detected with an ECL reagent kit (Millipore, OH, USA) and developed with an X-ray film. The density of protein bands was analyzed with ImageJ software.

### 2.7. Immunofluorescence

Immunofluorescence was performed on treated NSCs as we previously reported [15], with slight modification. In brief, the NSCs were fixed with 4% PFA and permeabilized with 0.1% Triton X-100. The fixed cells were incubated in 3% BSA-PBS solution to block the nonspecific proteins, incubated with anti-GRP78 and anti-phospho-p65 overnight at 4°C, and incubated with FITC/TRITC-conjugated secondary antibodies, for 2 hours at room temperature. The fluorescence data was collected with a fluorescence microscope and analyzed with ImageJ software.

### 2.8. Statistical Analysis

We evaluated the statistical significance according to independent-sample *t*-tests, followed by ANOVA tests for three or more groups of the data. Results are presented as means ± S.D. The value of *P* < 0.05 was considered to be significant.

## 3. Results

### 3.1. OGD/Reoxygenation-Induced NSC Apoptosis

The NSCs in this study were dissected from embryonic mouse (E14) brain, cultured as neurospheres in DMEM/F12 medium (containing B27, EGF, and bFGF), and identified with up to 95% of Nestin (the marker protein of NSCs) positive cell count, as we previously reported [15, 16].

**Figure 1:**
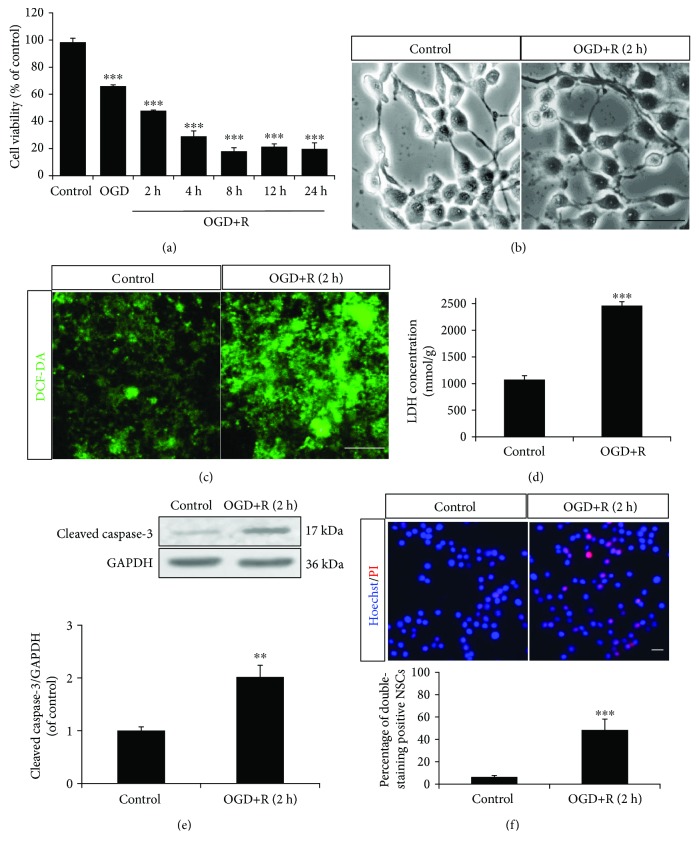
OGD/reoxygenation-induced NSC apoptosis. The NSCs cultured in the monolayer were subjected to OGD for 2 hours and reoxygenation for 2, 4, 8, 12, and 24 hours of induction. (a) Cell viability decreased relating to the duration of OGD/reoxygenation treatment through MTT assay. (b) The NSCs displayed an unhealthy morphology following OGD/reoxygenation induction. (c) The NSCs produced extra ROS with OGD/reoxygenation induction, according to DCF-DA fluorescence probe labelling. (d) The LDH of NSCs increased with OGD/reoxygenation induction, according to cellular LDH detection. (e) The protein expression of cleaved caspase-3 in NSCs was upregulated with OGD/reoxygenation induction, according to Western blotting. (f) OGD/reoxygenation treatment increased apoptosis of NSCs (Hoechst 33342/PI, double staining positive cells). ^∗∗^*P* < 0.01 and ^∗∗∗^*P* < 0.001 were considered to be significantly different between control and OGD or control and OGD+R groups. *n* = 3. Scale bar: 20 *μ*m.

The starved NSCs were transferred into monolayer culture for OGD/reoxygenation administration. According to morphology (Figure 1(b)), the OGD/reoxygenation-treated NSCs were detected with broken cytomembrane and axons and a wizened cell body, compared to healthy control NSCs with a bright and clear cell body and smooth and extended axons. Further, MTT assay demonstrated an exact NSC injury by OGD for 4 h with decreased cell viability from 98.4 ± 2.9% to 66.0 ± 1.1%; further injury was promoted by 2–24 h reoxygenation with cell viability which dropped to 47.9 ± 0.5%, 29.0 ± 4.0%, 18.0 ± 2.6%, 21.3 ± 2.1%, and 19.7 ± 4.5% (Figure 1(a)). With the moderate cell injury level (cell viability dropped to 47.9 ± 0.5%), OGD for 4 h followed by reoxygenation for 2 h was used for experimental administration in this study.

To explore the disturbing redox post-OGD/reoxygenation-induced injury, extra- and intracellular ROS and LDH productions were detected. According to DCF-DA probe fluorescence, the OGD/reoxygenation-treated NSCs produced obvious extra ROS, compared to nontreated cells (Figure 1(c)), while ELISA assay demonstrated a 2.3-fold higher concentration of LDH in OGD/reoxygenation-treated NSCs than that in control (Figure 1(d)).

Further, Hoechst 33342/PI and protein expression detection demonstrated significantly increased double staining and upregulated cleaved caspase-3 in OGD/reoxygenation-treated NSCs than that in control (Figures 1(e) and 1(f)). These results indicated that OGD/reoxygenation induced apoptosis in NSCs.

### 3.2. OGD/Reoxygenation-Triggered GRP78 Upregulation in NSCs

**Figure 2:**
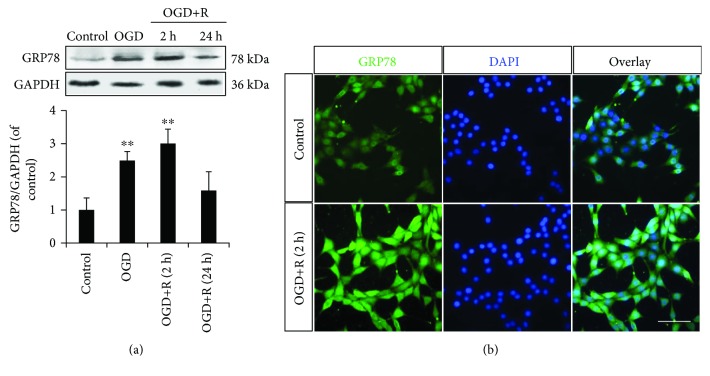
OGD/reoxygenation triggered GRP78 in NSCs. (a) The protein expression of GRP78 in NSCs was upregulated with OGD (2 h)/reoxygenation (2 h, 4 h), according to Western blotting. (b) Induced cytoplasm distribution of GRP78 around the nuclei in NSCs with OGD/reoxygenation treatment. ^∗∗^*P* < 0.01 was considered to be significantly different between control and OGD or control and OGD+R groups. *n* = 3. Scale bar: 20 *μ*m.

The protein expression of GRP78 demonstrated a significant upregulation with the OGD/reoxygenation administrations (Figures 2(a) and 2(b)). The NSCs injured by reoxygenation for 2 h peaked the GRP78 expression to 3-fold of that in nontreated NSCs, while the upregulation was shrunk to 1.6-fold of control in the OGD and 24 h reoxygenation-treated NSCs (Figure 2(a)). These results indicated a potential involvement of GRP78 in the signalling transduction of NSC apoptosis induced by OGD/reoxygenation.

### 3.3. Role of GRP78 in OGD/Reoxygenation-Induced NSC Apoptosis

To further explore the role of GRP78 in OGD/reoxygenation-induced NSC apoptosis, siRNA-transfected NSCs targeting at GRP78 were subjected to OGD/reoxygenation administration.

**Figure 3:**
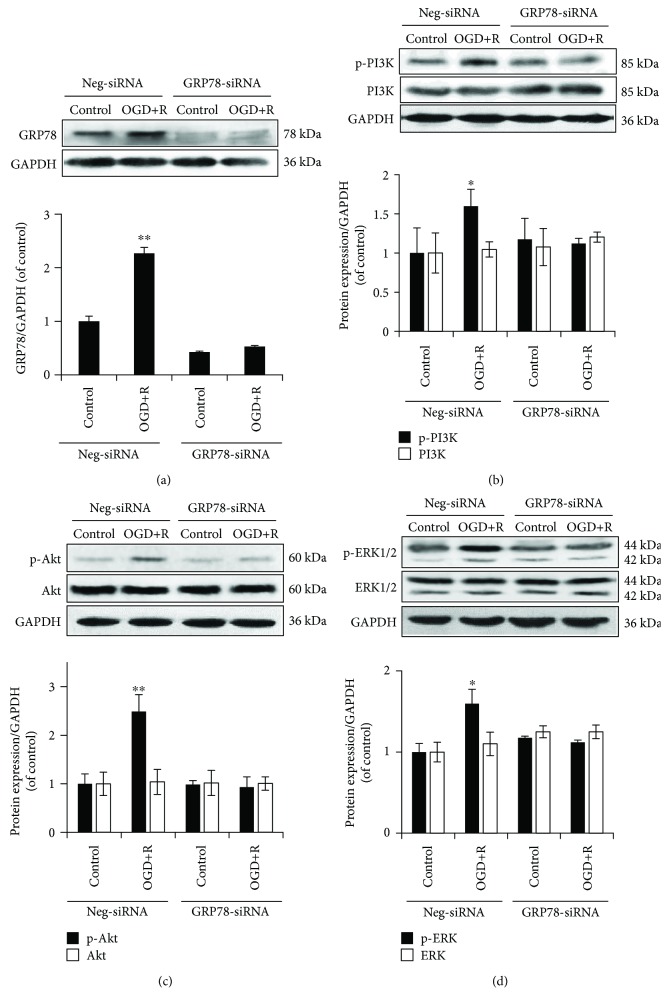
Role for GRP78 in OGD/reoxygenation-induced PI3K/Akt and ERK1/2 activations. (a) Protein expression of GRP78 in GRP78 knocked-down NSCs with OGD/reoxygenation induction, for siRNA transfecting efficiency determination. (b–d) Abolished phosphorylation of PI3K, Akt, and ERK1/2 by OGD+R induction in GRP78 knocked-down NSCs. GAPDH was used as an internal loading control. ^∗^*P* < 0.05 and ^∗∗^*P* < 0.01 were considered to be significantly different in Neg-siRNA-control versus Neg-siRNA-OGD+R, Neg-siRNA-control versus GRP78-siRNA-control, and GRP78-siRNA-control and GRP78-siRNA-OGD+R groups. *n* = 3.

The siRNA transfection efficiency was detected through the decreased protein expression of GRP78 in NSCs by 58 ± 6.8% and attenuated the induced upregulation of GRP78 by OGD/reoxygenation (Figure 3(a)). The result confirmed an efficiency knockdown of GRP78 and demonstrated a failed induction of GRP78 in NSCs by OGD/reoxygenation with decreased GRP78 expression.

Further protein detection demonstrated a significantly enhanced phosphorylation of PI3K, Akt, and ERK1/2 by OGD/reoxygenation in wild-type NSCs, while the induced enhancement was abolished by GRP78 knockdown (Figures 3(b)–3(d)), indicating a signalling transduction through GRP78 in OGD/reoxygenation-induced activation of PI3K/Akt and ERK1/2 in NSCs.

**Figure 4:**
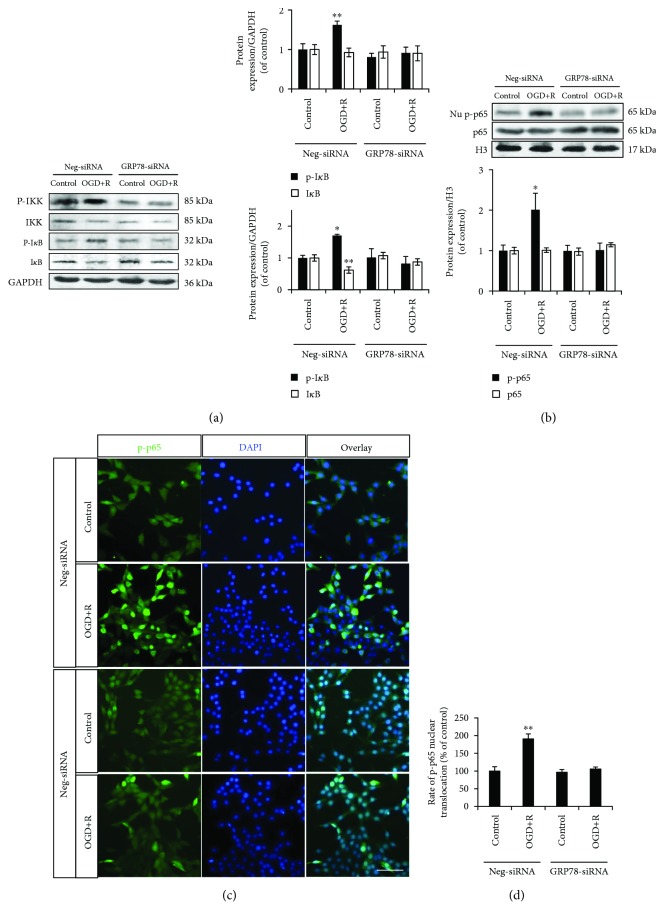
Role of GRP78 in OGD/reoxygenation-induced NF-*κ*B/p65 activations. (a, b) Abolished phosphorylation of IKK, I*κ*B, and p65 by OGD+R induction in GRP78 knocked-down NSCs. GAPDH was used as an internal loading control in whole cell lysate protein expression; H3 was used as an internal loading control in nuclear protein expression. (c, d) Abolished nuclear translocation of phospho-p65 by OGD+R induction in GRP78 knocked-down NSCs. ^∗^*P* < 0.05 and ^∗∗^*P* < 0.01 was considered to be significantly different in Neg-siRNA-control versus Neg-siRNA-OGD+R, Neg-siRNA-control versus GRP78-siRNA-control, and GRP78-siRNA-control and GRP78-siRNA-OGD+R groups. *n* = 3. Scale bar: 20 *μ*m.

The same abolishment of the enhanced phosphorylation was also detected on the upstreaming factors of NF-*κ*B/p65, IKK, and I*κ*B in GRP78 knocked-down NSCs (Figure 4(a)). As the downstreaming nuclear transfer factor, there was a significant augmentation of phosphorylated p65 in wild-type NSC nuclei (Figure 4(b)). Immunofluorescence demonstrated the consistently increased nuclear distribution of phosphorylated p65 (Figures 4(c) and 4(d)). However, in GRP78 knocked-down NSCs, both the enhanced phosphorylation and nuclear translocation of NF-*κ*B/p65 were abolished to normal level, which indicated a requirement of GRP78 in activation of NF-*κ*B/p65 by OGD/reoxygenation in NSCs (Figures 4(b)–4(d)).

**Figure 5:**
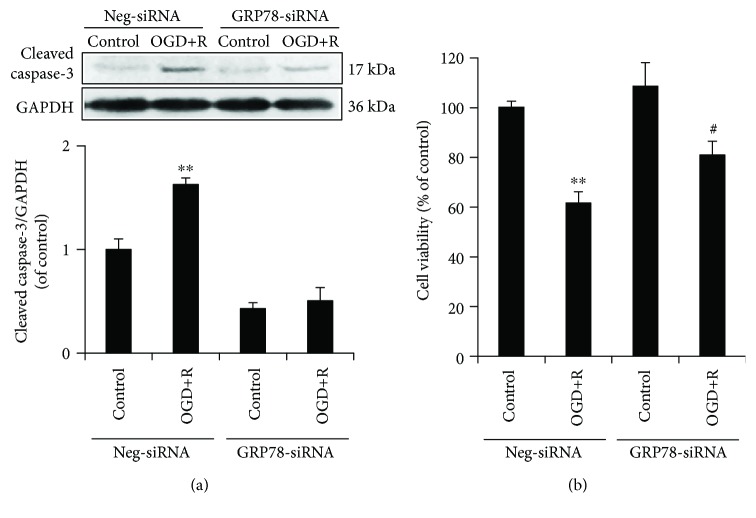
Role of GRP78 in OGD/reoxygenation-induced NSCs. (a) Abolished upregulation of cleaved caspase-3 by OGD+R in GRP78 knocked-down NSCs. (b) Attenuated cellular injury by OGD+R in GRP78 knocked-down NSCs. ^∗∗^*P* < 0.01 was considered to be significantly different between Neg-siRNA-control and Neg-siRNA-OGD+R groups; ^#^*P* < 0.05 was considered to be significantly different between GRP78-siRNA-control versus GRP78-siRNA-OGD+R groups. *n* = 3.

Following the signalling pathway activation, we further investigated the NSC apoptosis and viability with a lower level of GRP78. The results demonstrated that in GRP78 knocked-down NSCs, the enhanced protein expression of cleaved caspase-3 by OGD/reoxygenation was abolished (Figure 5(a)), while the attenuated cell viability was improved, but still statistically significant (Figure 5(b)).

Taken together, the results indicated a requirement of GRP78 in OGD/reoxygenation-induced NSC apoptosis through PI3K/Akt, ERK1/2, and NF-*κ*B/p65 activation.

### 3.4. Cross Talk among GRP78, PI3K/Akt, ERK1/2, and NF-*κ*B/p65 in OGD/Reoxygenation-Induced NSCs

To further explore the cross talk among GRP78, PI3K/Akt, ERK1/2, and NF-*κ*B/p65 in OGD/reoxygenation-induced NSC apoptosis, we, respectively, applied the inhibitors of PI3K/Akt, ERK1/2 and NF-*κ*B/p65, LY294002, SCH77298, and JSH23.

**Figure 6:**
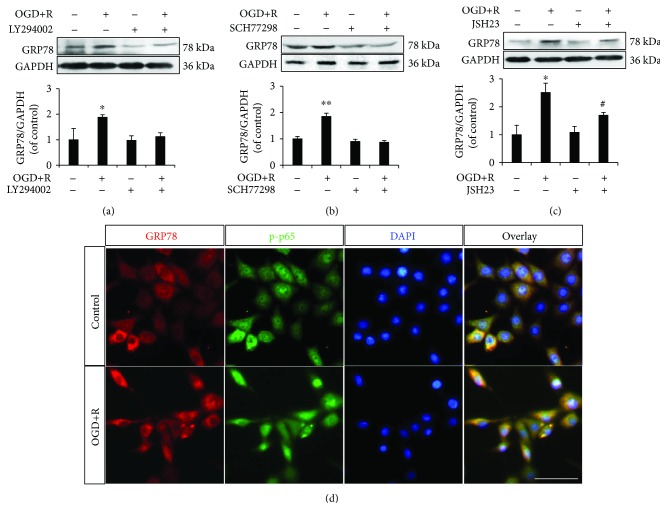
Role of PI3K/Akt, ERK1/2, and NF-*κ*B/p65 in OGD/reoxygenation-triggered GRP78 upregulation. (a) Abolished upregulation of GRP78 by OGD+R in LY294002-treated NSCs; (b) abolished upregulation of GRP78 by OGD+R in SCH77298-treated NSCs; (c) upregulated GRP78 by OGD+R in either control or JSH23-pretreated NSCs; (d) cellular distribution and coactivation of GRP78 and phospho-p65 in OGD+R-induced NSCs. ^∗^*P* < 0.05 and ^∗∗^*P* < 0.01 were considered to be significantly different between control and OGD+R groups; ^#^*P* < 0.05 was considered to be significantly different between JSH23 and JSH23+OGD+R groups. *n* = 3. Scale bar: 20 *μ*m.

According to the protein expression, using LY294002 and SCH77298, respectively, abolished the upregulated GRP78 by OGD/reoxygenation treatment (Figures 6(a) and 6(b)), which drew a signalling transduction through PI3K/Akt or ERK1/2 to GRP78.

Although JSH23 was also detected attenuating the induced GRP78 slightly, the effect was not statistically significant (Figure 6(c)), which disconfirmed the signalling transduction from NF-*κ*B/p65 to GRP78.

Taking together the results from siRNA transfection and inhibitor applications, it indicated two bidirectional signalling transductions of GRP78-PI3K/Akt and GRP78-ERK1/2 and a one-way signalling transduction through GRP78 to NF-*κ*B/p65 in response to OGD/reoxygenation in NSCs.

To further examine the relationship between GRP78 and NF-*κ*B/p65, we investigated the distribution of the two signalling proteins in NSCs by OGD/reoxygenation. Immunofluorescence on GRP78/phospho-p65 demonstrated the enhanced signal of GRP78 in the cytoplasm around the nuclei and of phospho-p65 in nuclei by OGD/reoxygenation treatment (Figure 6(d)), which confirmed the coactivation of GRP78 and NF-*κ*B/p65 in OGD/reoxygenation-induced NSCs.

## 4. Discussion

In the central nervous system (CNS), all types of neural cells receive influence from the cellular ischemia/reperfusion cascade of oxidative stress, calcium disturbance, inflammation, immune reaction, DNA damage, and so on, induced by ischemic stroke [18]. The ischemia/reperfusion cascade not only damages neurons and oligodendrocytes and activates astrocytes and microcytes but also induces the NSCs located in SVZ and SGZ to migrate to the lesion point, produce neural growth/trophic factors for injured cell rescue, and differentiate into mature neural cells for damaged cell replacement [19–21]. However, several studies have reported that a huge part of the local NSCs were damaged to apoptosis and death by the ischemia/reperfusion cascade before migration and differentiation, resulting to a failed neuroregeneration [16, 19, 22]. Our result with the *in vitro* OGD/reoxygenation-induced NSCs demonstrated the induced oxidative stress and apoptosis, which is in consistence with these previous reports. In concern of the essential role of NSCs in neuroregeneration post ischemia stroke, it is a critical strategy to explore the mechanism to promote NSC survival from apoptosis following ischemia/reperfusion damage.

Our previous study has reported about oxidative stress-induced apoptosis and the involved HSP90/NF-*κ*B/p65 signalling mechanism in NSCs [15]. In contrast, besides the canonical mechanisms, such as oxidative stress, ER stress has less been reported in OGD/reoxygenation-induced NSC apoptosis [23, 24]. ER serves as an organelle to fold, facilitate, and transport the newly synthesized proteins, with the collaboration of several ER chaperone proteins, such as GRP78 [25]. The properly folded proteins will be transported by ER to the Golgi apparatus, leaving the unfolded and misfolded proteins to be accumulated in ER. As long as the unfolded proteins overloaded in ER responding to hypoxia, disturbance of glucose metabolism, and other disorders, ER stress emerges and GRP78 is sharply augmented to attenuate the pathological situation. Due to the sensitivity to hypoxia, glucose, and ion disturbance, ER stress is mostly studied in cancer cells [14]. Reports have documented that ER stress is correlated with cancer cell activation, and an ER chaperone protein, GRP78, is enhanced in cancer cell proliferation and apoptosis through MAPKs, PI3K/Akt, and NF-*κ*B signalling pathways [26, 27]. GRP78 has been postulated as a potential target for cancer cure and a potential prognostic marker [14, 28]. In spite of some studies on the involvement of ER stress and GRP78 in neurons and astrocytes against neurodegeneration, neither the role of GRP78 in NSC survival nor the signalling transduction during the procedure has yet been elucidated clearly. In this study, we have detected a time-related upregulation of GRP78 in the NSC cytoplasm induced by OGD/reoxygenation and an activation of PI3K/Akt, ERK1/2, and NF-*κ*B/p65. These data indicated the participation of GPR78, PI3K/Akt, ERK1/2, and NF-*κ*B/p65 in NSC apoptosis induced by OGD/reoxygenation.

We further investigated the cross talk among GRP78, PI3K/Akt, ERK1/2, and NF-*κ*B/p65 in OGD/reoxygenation-induced NSCs. PI3K/Akt and ERK1/2 have always been documented as essential signalling pathways on cell survival in response to a series of physiological and pathological induction, as well as exogenous stimulations, via downstreaming signalling factors, such as NF-*κ*B/p65 and GSK-3*β*. A study on cancer cells has reported activated PI3K/Akt and ERK1/2 following enhanced GRP78, indicating ER stress as a cause of PI3K/Akt and ERK1/2 activation [14, 26]. Our results using GRP78 knocked-down NSCs demonstrated a failed activation of PI3K/Akt and ERK1/2 following OGD/reoxygenation, while, respectively, blockage of PI3K/Akt or ERK1/2 by the inhibitor GRP78 was detected to have failed to respond to OGD/reoxygenation stimulation. These results indicated the bidirectional regulations of GRP78-PI3K/Akt and GRP78-ERK1/2, which both contributed in promoting NSCs against OGD/reoxygenation-induced apoptosis.

NF-*κ*B/p65 has always been documented as a key nuclear transcription factor against stimulation and stress and has been recognized as a downstreaming mediator of GRP78, PI3K/Akt, and ERK1/2 in cancer cells; the computational simulation noted the GRP78-NF-*κ*B-binding interaction working as a potential neuroprotective pathway in brain injury [26, 29]. In our study on OGD/reoxygenation-induced NSCs, the result demonstrated an abolished activation of IKK, I*κ*B, and p65 in the absence of GRP78, while GRP78 was always triggered with or without NF-*κ*B blockage. Although we assumed a potential bidirectional regulation between GRP78 and NF-*κ*B as GRP78-PI3K/Akt and GRP78-ERK1/2, the result indicated a one-way signalling transduction through GRP78 to NF-*κ*B/p65, which is different from that in GRP78-PI3K/Akt and GRP78-ERK1/2. Furthermore, the increased cytoplasm distribution of GRP78 and nuclear distribution of NF-*κ*B/p65 according to two-channel immunofluorescence have further confirmed the coactivation of GRP78 and NF-*κ*B/p65.

According to other researches, there are other kinds of signalling pathways interacting with GRP78, besides PI3K/Akt, ERK1/2, and NF-*κ*B/p65. When ER stress happens, GRP78 composes a complex with inositol-requiring enzyme 1 (IRE1), PKR-like ER kinase (PERK), and activating transcription factor 6 (ATF6) [30]. Dissociated from the GRP78 complex under ER stress, IRE1 is triggered to promote TNF receptor-associated factor 2 (TRAF2) activation and conjugate to the TRAF2-ASK complex, after which it induces the activation of JNK signalling. The activating cascades result in the Bax/Bak-dependent apoptosis or autophagy [30, 31]. When GRP78 releases from the complex, PERK is stimulated to promote the phosphorylation of nuclear factor erythroid 2-related factor 2 (NRF2), which plays an antioxidative role in cancer cells [32, 33]. In addition, phosphorylated PERK by GRP78 plays a role in obesity and diabetes through the eIF2*α*-ATF4-CHOP pathway [34]. At the same time, PERK and IRE1 in turn modulate GRP78 activation [35]. It was evidenced that GRP78 induces the activation of MAPKs, resulting in autophagy in breast cancer [36]. GRP78 is also reported to suppress the transforming growth factor-*β* (TGF-*β*) pathway, which promotes cell survival and growth [37], and it protects against ER stress-mediated apoptosis in chondrocyte differentiation through inhibition of caspase-3, caspase-12, CHOP, and p-JNK signalling [13, 34]. Another previous study reported that GRP78 led to the activation of PAK-2 and LIMK1 to accelerate the metastasis in prostate cancer [38]. However, the role of GRP78 and the signalling cross talk have rarely been reported in NSC survival.

**Figure 7:**
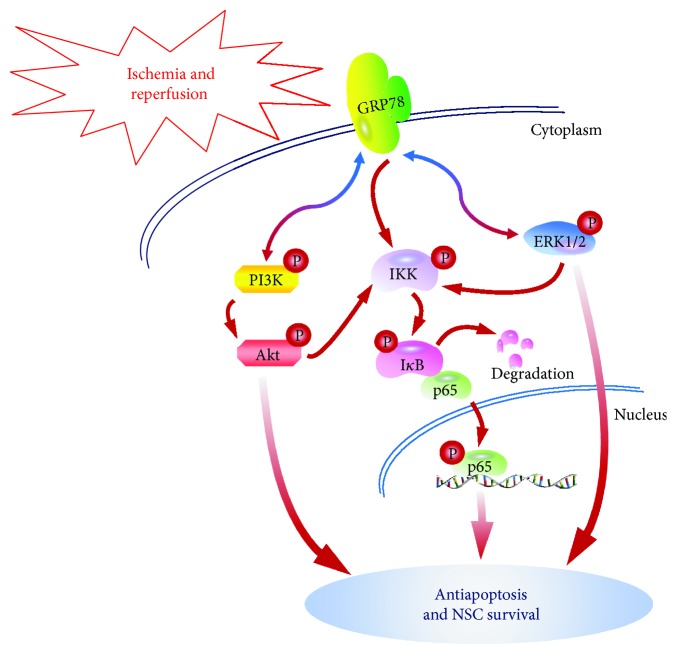
Diagram of GRP78-mediated NSC survival from OGD/reoxygenation-induced apoptosis through PI3K/Akt, ERK1/2, and NF-*κ*B/p65. GRP78 in NSCs is triggered by OGD/reoxygenation. The signalling is transmitted through PI3K/Akt, ERK1/2, and NF-*κ*B/p65 to cellular antiapoptosis and survival. Meanwhile, the activated PI3K/Akt and ERK1/2 in turn improve GRP78 upregulation. In summary, the bidirectional signalling transductions of GRP78-PI3K/Akt and GRP78-ERK1/2 and the one-way signalling transductions through GRP78 to NF-*κ*B/p65 would contribute essentially to NSC survival from OGD/reoxygenation-induced apoptosis.

In summary of the present study, we have drawn a signalling network in OGD/reoxygenation-induced NSC apoptosis. GRP78 of NSCs is triggered in response to OGD/reoxygenation stimulation and conducted the pathological signalling to PI3K/Akt, ERK1/2, and NF-*κ*B/p65 activation, while the activated PI3K/Akt and ERK1/2 in turn promoted the induced GRP78 upregulation, which forms the bidirectional regulations of GRP78-PI3K/Akt and GRP78-ERK1/2 to promote NSC survival. However, it is a one-way signalling transduction through GRP78 to NF-*κ*B/p65, which also leads to antiapoptosis (Figure 7). Taken together, GRP78 mediates signalling transduction through PI3K/Akt, ERK1/2, and NF-*κ*B/p65, which would rescue NSCs from OGD/reoxygenation-induced apoptosis.

## Abbreviations

Akt: Protein kinase B (PKB)
bFGF: Basic fibroblast growth factor
BSA: Bovine serum albumin
DMEM: Dulbecco's modified eagle medium
DMSO: Dimethyl sulphoxide
ECL: Enhanced chemiluminescence
EGF: Epidermal growth factor
ERK1/2: Extracellular signal-regulated kinase 1/2
GAPDH: Glyceraldehyde phosphate dehydrogenase
HSP70: Heat shock protein 70
IKK: I*κ*B kinase
I*κ*B: Inhibitory protein of NF-*κ*B
LDH: Lactate dehydrogenase
MAPKs: Mitogen-activated protein kinases
MTT: 3-(4,5-Dimethylthiazol-2-yl)-2,5-diphenyltetrazolium bromide
NF-*κ*B: Nuclear factor-*κ*B
PI3K: Phosphatidylinositol 3-kinase
PVDF: Polyvinylidene fluoride
PI: Propidium iodide
ROS: Reactive oxygen species
TBST: Tris-buffered saline-Tween 20.
